# Synthesis, *in vitro* biological assessment, and molecular docking study of benzimidazole-based thiadiazole derivatives as dual inhibitors of α-amylase and α-glucosidase

**DOI:** 10.3389/fchem.2023.1125915

**Published:** 2023-05-05

**Authors:** Shoaib Khan, Shahid Iqbal, Muhammad Taha, Rafaqat Hussain, Fazal Rahim, Mazloom Shah, Nasser S. Awwad, Hala A. Ibrahium, Mohammed Issa Alahmdi, Ayed A. Dera, Hayat Ullah, Ali Bahadur, Samar O. Aljazzar, Eslam B. Elkaeed, Muhammad Rauf

**Affiliations:** ^1^ Department of Chemistry, Hazara University, Mansehra, Pakistan; ^2^ Department of Chemistry, School of Natural Sciences (SNS), National University of Science and Technology (NUST), Islamabad, Pakistan; ^3^ Department of Clinical Pharmacy, Institute for Research and Medical Consultations (IRMC), Imam Abdulrahman Bin Faisal University, Dammam, Saudi Arabia; ^4^ Department of Chemistry, Abbottabad University of Science and Technology (AUST), Abbottabad, Pakistan; ^5^ Department of Chemistry, King Khalid University, Abha, Saudi Arabia; ^6^ Department of Biology, Nuclear Materials Authority, El Maadi, Egypt; ^7^ Department of Semi Pilot Plant, Nuclear Materials Authority, El Maadi, Egypt; ^8^ Department of Chemistry, Faculty of Science, University of Tabuk, Tabuk, Saudi Arabia; ^9^ Department of Clinical Laboratory Sciences, College of Applied Medical Sciences, King Khalid University, Abha, Saudi Arabia; ^10^ Department of Chemistry, University of Okara, Okara, Punjab, Pakistan; ^11^ Department of Chemistry, College of Science and Technology, Wenzhou-Kean University, Wenzhou, China; ^12^ Department of Chemistry, College of Science, Princess Nourah bint Abdulrahman University, Riyadh, Saudi Arabia; ^13^ Department of Pharmaceutical Sciences, College of Pharmacy, AlMaarefa University, Riyadh, Saudi Arabia; ^14^ Department of Chemistry, School of Science, University of Management and Technology, Lahore, Pakistan

**Keywords:** synthesis, benzimidazole, thiadiazol, α-amylase, α-glucosidase, SAR and molecular docking, α-glucosidase

## Abstract

The clinical significance of benzimidazole-containing drugs has increased in the current study, making them more effective scaffolds. These moieties have attracted strong research interest due to their diverse biological features. To examine their various biological significances, several research synthetic methodologies have recently been established for the synthesis of benzimidazole analogs. The present study aimed to efficiently and quickly synthesize a new series of benzimidazole analogs. Numerous spectroscopic techniques, including ^1^H-NMR, ^13^C-NMR, and HREI-MS, were used to confirm the synthesized compounds. To explore the inhibitory activity of the analogs against α-amylase and α-glucosidase, all derivatives (**1–17**) were assessed for their biological potential. Compared to the reference drug acarbose (IC_50_ = 8.24 ± 0.08 µM), almost all the derivatives showed promising activity. Among the tested series, analog **2** (IC_50_ = 1.10 ± 0.10 & 2.10 ± 0.10 µM, respectively) displayed better inhibitory activity. Following a thorough examination of the various substitution effects on the inhibitory capacity of α-amylase and α-glucosidase, the structure-activity relationship (SAR) was determined. We looked at the potential mechanism of how active substances interact with the catalytic cavity of the targeted enzymes in response to the experimental results of the anti-glucosidase and anti-amylase. Molecular docking provided us with information on the interactions that the active substances had with the various amino acid residues of the targeted enzymes for this purpose.

## Introduction

**FIGURE 1:**
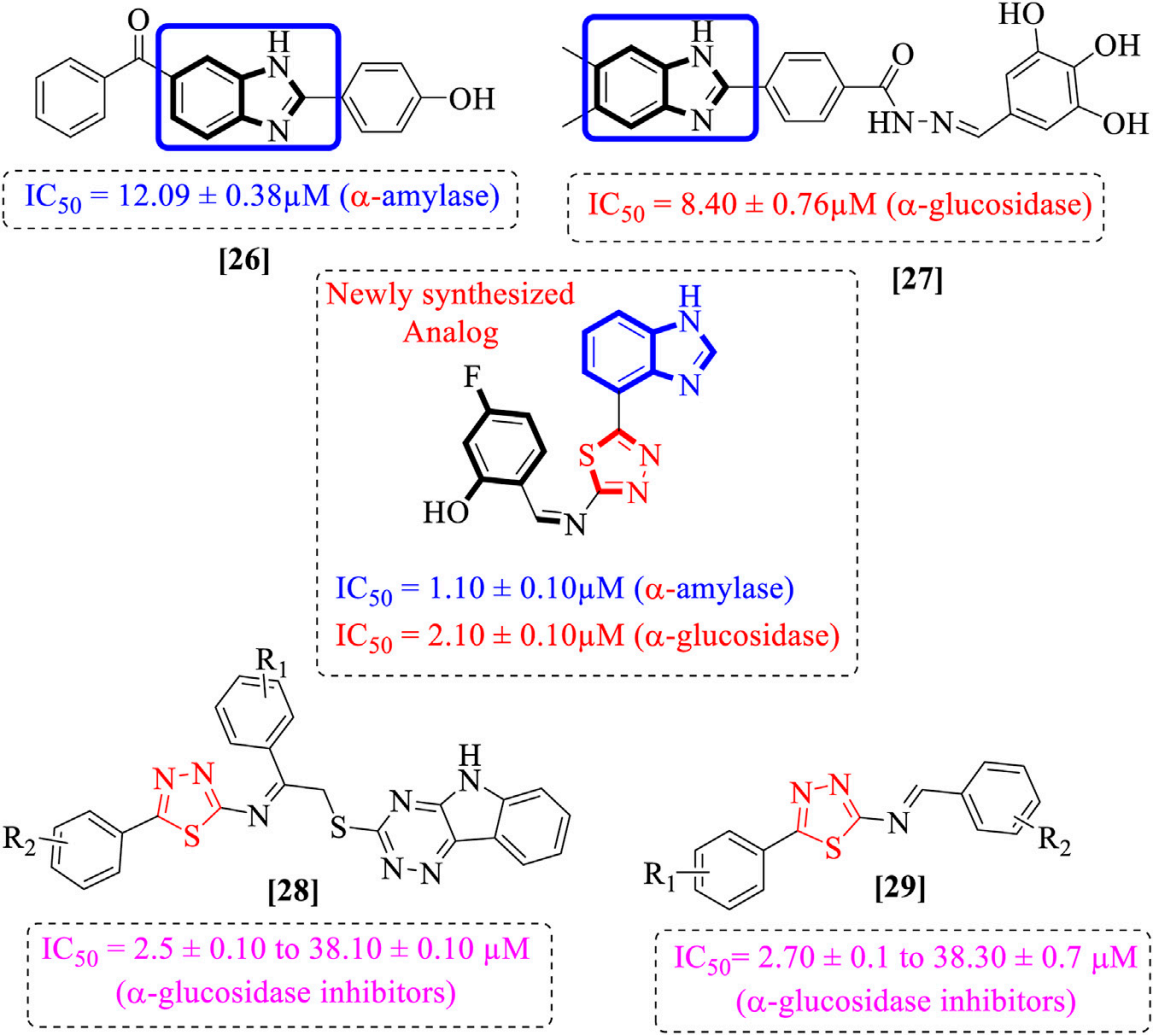
Rationale of the current study.

**FIGURE 2:**
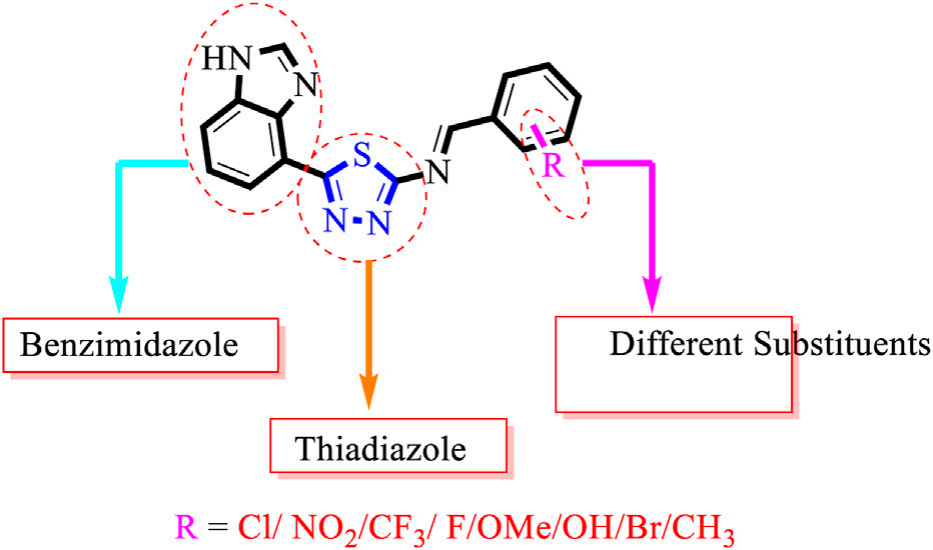
General structures of the target compounds showing different structural fragments.

The natural metabolism of proteins, lipids, and carbohydrates is impaired by the chronic illness of diabetes mellitus. Additionally, diabetes leads to metabolic changes that cause hyperglycemia (American Diabetes Association, 2013). The prevalence of diabetes has increased globally by 4.4% since 2000 (2.8%), and more than 366 million people will have diabetes worldwide by 2030 (Wild et al., 2004). Sulfonylureas, biguanides, and α-glucosidase inhibitors are only a few of the synthetic oral hypoglycemic drugs used to lower high blood glucose levels. Unfortunately, due to their extended use, the side effects include hypoglycemia, headaches, nausea, and dizziness (Edwin et al., 2006; Chaudhury et al., 2017). Thus, owing to these detrimental impacts, finding novel, effective, and safer drugs is of the utmost importance (Es-Safi et al., 2020). Researchers are currently focusing on heterocyclic compounds due to their potential effectiveness, availability, and generally fewer adverse effects. The present study also evaluates heterocyclic compounds such as benzimidazole-based thiadiazole derivatives. Benzimidazole rings have attracted considerable attention in comparison to other heterocyclic compounds due to their biological significance as the ring is frequently referred to as “privileged”. Researchers are particularly interested in the structure of benzimidazole; The chemistry of their ligands was first described in 1950 (Nofal et al., 2011; Walia et al., 2011). The diverse biological applications of benzimidazole and chemically related substances, such as purine, have also been reported. The key component of the complex, the benzimidazole ring, which is present in vitamin B-12 as 5,6-dimethyl-1-(a-D-ribofuranosyl), was also identified in vitamin B-12 in 1948 (Bonnett, 1963). Metronidazole, thiabendazole, misonidazole, omeprazole, astemizole, clotrimazole, azomycin, cimetidine, and antihistamines are examples of comparable products used in the veterinary, pharmaceutical, and agricultural industries (Al-Muhaimeed, 1997). Drugs containing the benzimidazole moiety have a wide range of pharmacological properties, including bactericidal (Carcanague et al., 2002; Shehab and Mansour, 2021), analgesic (Demirayak et al., 2005; Aghatabay et al., 2007), fungicidal (Solel, 1970; Lezcano et al., 2002), antiviral (Tewari and Mishra, 2006; Tonelli et al., 2008), anti-malarial (Worachartcheewan et al., 2013), HIV-1 infectivity inhibition (Gardiner et al., 1995; Kanwal et al., 2019), AChE and BuChE inhibition (Dinparast et al., 2021; Türkan, 2021), antioxidant (Ayhan-Kilcigil et al., 2004; Usta et al., 2015), and antileishmanial (Mayence et al., 2008) effects. In the field of current medicinal chemistry, researchers have shown that the hybridization of two or more molecules with various bioactive structural motifs is an efficient method for creating novel chemical entities with improved pharmacological properties. In response to the aforementioned findings and ongoing research on the synthesis of novel benzimidazole (Zawawi et al., 2016; Aroua et al., 2021) and thiadiazole (Javid et al., 2018; Khan et al., 2022a) as α-glucosidase agents, we designed and synthesized a series of novel chemical entities together with benzimidazole and thiadiazole structural motifs (Figure 2) to generate potent α-amylase and α-glucosidase agents (Figure 1).

## Results and discussion

### Chemistry

**SCHEME 1:**
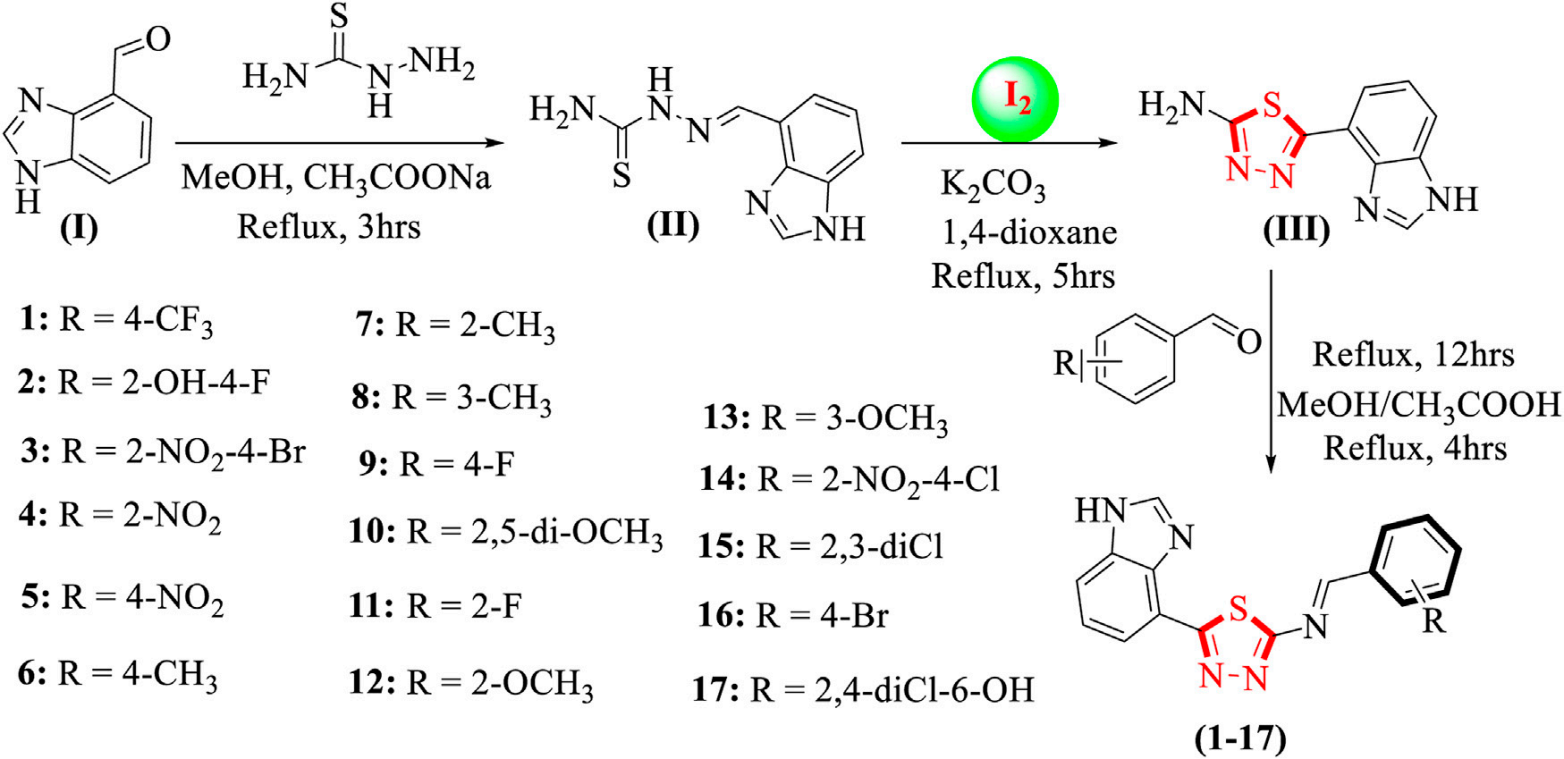
Synthesis of the benzimidazole-based thiadiazole derivatives (**1-17**).

The approach taken in the synthesis of thiadiazole derivatives based on benzimidazoles. By treating benzimidazole having an aldehyde group (I) and thiosemicarbazide in methanol and refluxing the reaction mixture for about 3 hours while sodium acetate waspresent, all the synthesized products were obtained. Schiff base was then produced as an intermediate (II). Iodine and potassium carbonate in 1,4-dioxane were then added and refluxed for 5 h to produce a benzimidazole-based thiadiazole containing an amine group (**III**), which was further mixed in methanol and refluxed for 4 h along with different substituted aldehydes in the presence of acetic acid to obtain benzimidazole-based thiadiazole derivatives (**1-17**), as shown in Scheme 1. Several spectroscopic methods, including 1H-NMR, 13C-NMR, and HREI-MS, were used to confirm the exact structures of all the synthesized analogues. The proton NMR spectrum of compound **14** indicated that the most de-shielded proton attached to the nitrogen of benzimidazole nitrogen requires a lower applied magnetic field to achieve resonance, resulting in a singlet at chemical shift values of 10.23 ppm. Two more singlets were also observed: one for the (HC = N) proton resonating at a chemical shift of 7.39 ppm and the other for benzimidazole (2-position) proton resonating at a chemical shift value of 7.30 ppm. A doublet of doublets with chemical shift values resonati Qn 1g 1 at 8.50 ppm are created when the proton at position 5 of the benzimidazole ring combines with the ortho-proton next to it to form a doublet, which is further split by its meta-proton. While a proton in the 7-position of benzimidazole appeared as a doublet with a chemical shift value of 7.47 ppm, a proton in the 6-position simultaneously bonded to its surrounding ortho-protons and appeared as a triplet with a chemical shift value of 7.42 ppm. The aromatic ring is further joined to three more protons. Protons at positions 3 and 6 of the aromatic ring form doublets when they couple with neighboring protons, and the proton at position 5 of the aryl ring produces a doublet of doublet that may be seen at chemical shift values of 8.10 ppm.

### Spectral analysis

The spectral analyses of the synthesized analogs are included in the Supplementary Information.

## *In vitro* α-amylase and α-glucosidase inhibitory activities

Diabetes mellitus (DM) is a chronic disorder that occurs due to the adverse effects of α-amylase and α-glucosidase enzymes. Various drugs are used to inhibit these enzymes but have risks for serious complications. To overcome these complications, researchers have focused on heterocyclic compounds to synthesize effective inhibitors for the treatment of DM. Among these heterocyclic compounds are benzimidazole-based thiadiazoles. The present study synthesized 17 benzimidazole-based thiadiazole compounds and evaluated their effectiveness against α-amylase and α-glucosidase enzymes. Due to different substituents on the aromatic ring (represented by “R”), these compounds showed a range of inhibitions, with IC_50_ values ranging from 1.10 ± 0.10 to 24.20 ± 0.40 (α-amylase) and 2.10 ± 0.10 to 26.10 ± 0.10 µM (α-glucosidase).

### Structure–activity relationship (SAR)

Inhibitory profiles can change depending on the substituent nature, number, and position. Substituent location on the *para, ortho*, and *meta-*positions of the aromatic ring and the nature of the substituents determine the functionality of electron-withdrawing groups (EWGs) or electron-donating groups (EDGs). Moreover, the number of substituents indicates the presence of one or more substituents (same or different). The tested analogs were compared by placing the same substituents at different positions of the aromatic ring (Figure 2).

Comparison of analogs with substituted moieties against both α-amylase and α-glucosidase enzymes showed different inhibitory profiles. Nitro-substituted analogs **3** (IC_50_ = 22.90 ± 0.10 and 24.60 ± 0.20 µM), **4** (IC_50_ = 6.80 ± 0.20 and 7.40 ± 0.30 µM), **5** (IC_50_ = 4.80 ± 0.10 and 5.70 ± 0.20 µM), and **14** (IC_50_ = 8.10 ± 0.30 and 9.30 ± 0.40 µM) were compared with the standard drug acarbose (IC50 = 4.16 ± 0.17 and 5.30 ± 0.12 µM, respectively). Among these nitro group analogs, analog **5** showed remarkable potential, while the others showed good to moderate activity, possibly due to the presence of nitro moiety at the *para-*position of the aromatic ring, which produces a strong tendency for hydrogen bonding. The remaining analogs showed comparable activity to standard drugs. The lower potential of analog **3** might be due to the presence of the bulky bromo group at the *para-*position of the ring.

Replacement of the nitro group by a chloro group at different positions changed the ring activity profiles, possibly due to the nucleophilic characteristic of chlorine, which creates strong interaction with the active sites of enzymes. Analogs **15** (IC_50_ = 5.40 ± 0.050 and 6.60 ± 0.10 µM) and **17** (IC_50_ = 2.16 ± 0.50 and 2.30 ± 0.60 µM respectively) showed better inhibitory profiles than nitro-substituted analogs. The change in the activity profile also depends on the number of substituents. Analog **15** bears two chloro groups at the *ortho* and *meta*-positions, while analog **17** has two chloro groups at the *ortho-* and *para-*positions and a hydroxyl group at the *ortho-*position. Thus, the number and nature of the substituents also increased the activity in the case of chloro groups. The hydroxyl group increased the analog efficacy through hydrogen binding; thus, analog **17** was much more potent than analog **15** and exhibited a two-fold better activity profile than the standard drug acarbose.

Likewise, fluoro substituents replacing the chloro group further increase the biological potential of analogs that might be smaller in size and can make strong hydrogen bonds with the active sites of enzymes. Among fluoro-substituted analogs **2** (IC_50_ = 1.10 ± 0.10 and 2.10 ± 0.10 µM), **9** (IC_50_ = 2.40 ± 0.010 and 3.90 ± 0.10 µM), and **11** (IC_50_ = 3.18 ± 0.10 and 4.70 ± 0.10 µM, respectively), the differences in inhibitory profiles were due to the position of the fluoro group. Analog **2** was the most potent analog among the series due to both fluoro and hydroxyl moieties (*para* and *ortho-*positions, respectively) on the ring leading to the formation of stronger hydrogen bonds with the active sites of enzymes. In addition, the trifluoro-substituted analog **1** (IC_50_ = 2.20 ± 0.10 and 2.80 ± 0.20 µM, respectively) also showed two-fold better results than acarbose.

Similarly, the methyl/methoxy/and bromo-substituted analogs (**3,6-8, 10, 12, 13,** and **16**) showed at least comparable activity to the standard drug acarbose. The bulky nature of bromine and steric hindrance by the methyl moiety decrease the analog potentials. Varied ranges of inhibitory profiles were exhibited by these analogs (Table 1).

**TABLE 1:** α-Amylase and α-glucosidase inhibitory activities of benzimidazole-based thiadiazole derivatives **(1-17)**.

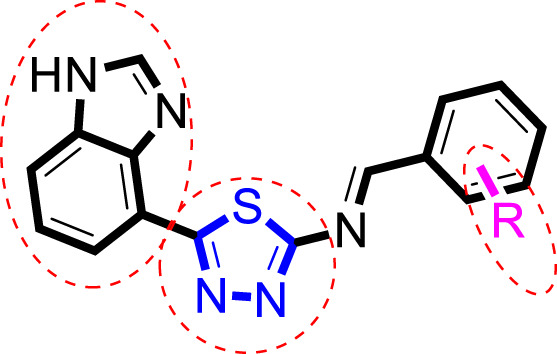			
**S. No.**	**R**	α-**Amylase IC**_**50**_ **(*µ*M ±SEM)**	α-**Glucosidase IC**_**50**_ **(*µ*M ±SEM)**
**1**	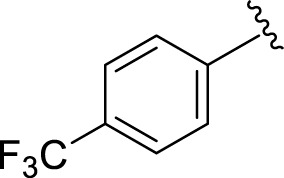	2.20 ± 0.10	2.80 ± 0.20
**2**	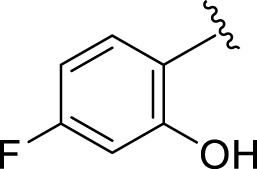	1.10 ± 0.10	2.10 ± 0.10
**3**	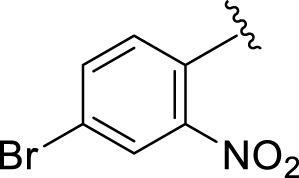	22.90 ± 0.10	24.60 ± 0.20
**4**	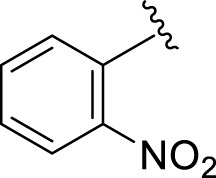	6.80 ± 0.20	7.40 ± 0.30
**5**	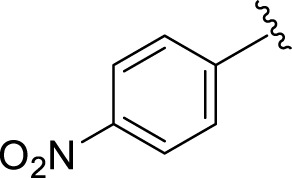	4.80 ± 0.10	5.70 ± 0.20
**6**	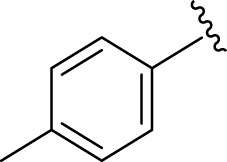	13.80 ± 0.20	15.30 ± 0.10
**7**	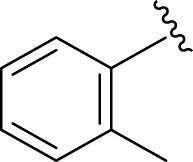	18.70 ± 0.20	19.90 ± 0.10
**8**	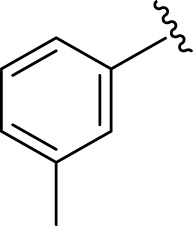	19.60 ± 0.20	21.60 ± 0.30
**9**	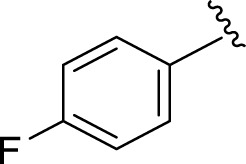	2.40 ± 0.010	3.90 ± 0.10
**10**	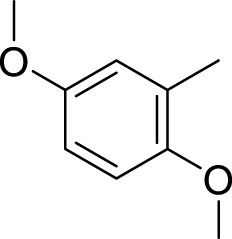	14.35 ± 0.50	14.50 ± 0.50
**11**	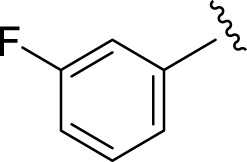	3.18 ± 0.10	4.70 ± 0.10
**12**	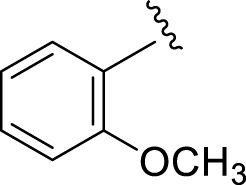	14.30 ± 0.20	15.40 ± 0.30
**13**	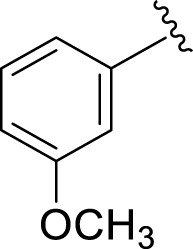	15.70 ± 0.30	17.20 ± 0.30
**14**	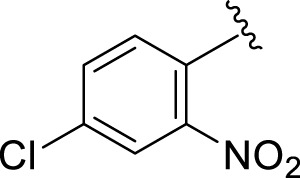	8.10 ± 0.30	9.30 ± 0.40
**15**	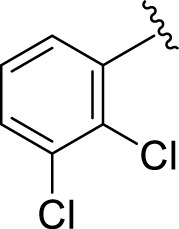	5.40 ± 0.050	6.60 ± 0.10
**16**	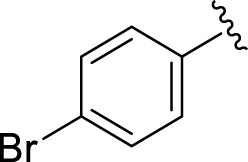	24.20 ± 0.40	26.10 ± 0.10
**17**	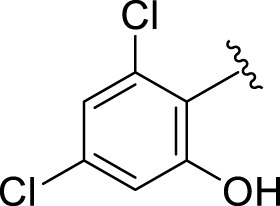	2.16 ± 0.50	2.30 ± 0.60
**Standard drug acarbose**		**4.16 ± 0.17**	**5.30 ± 0.12**

### Molecular docking studies

**FIGURE 3:**
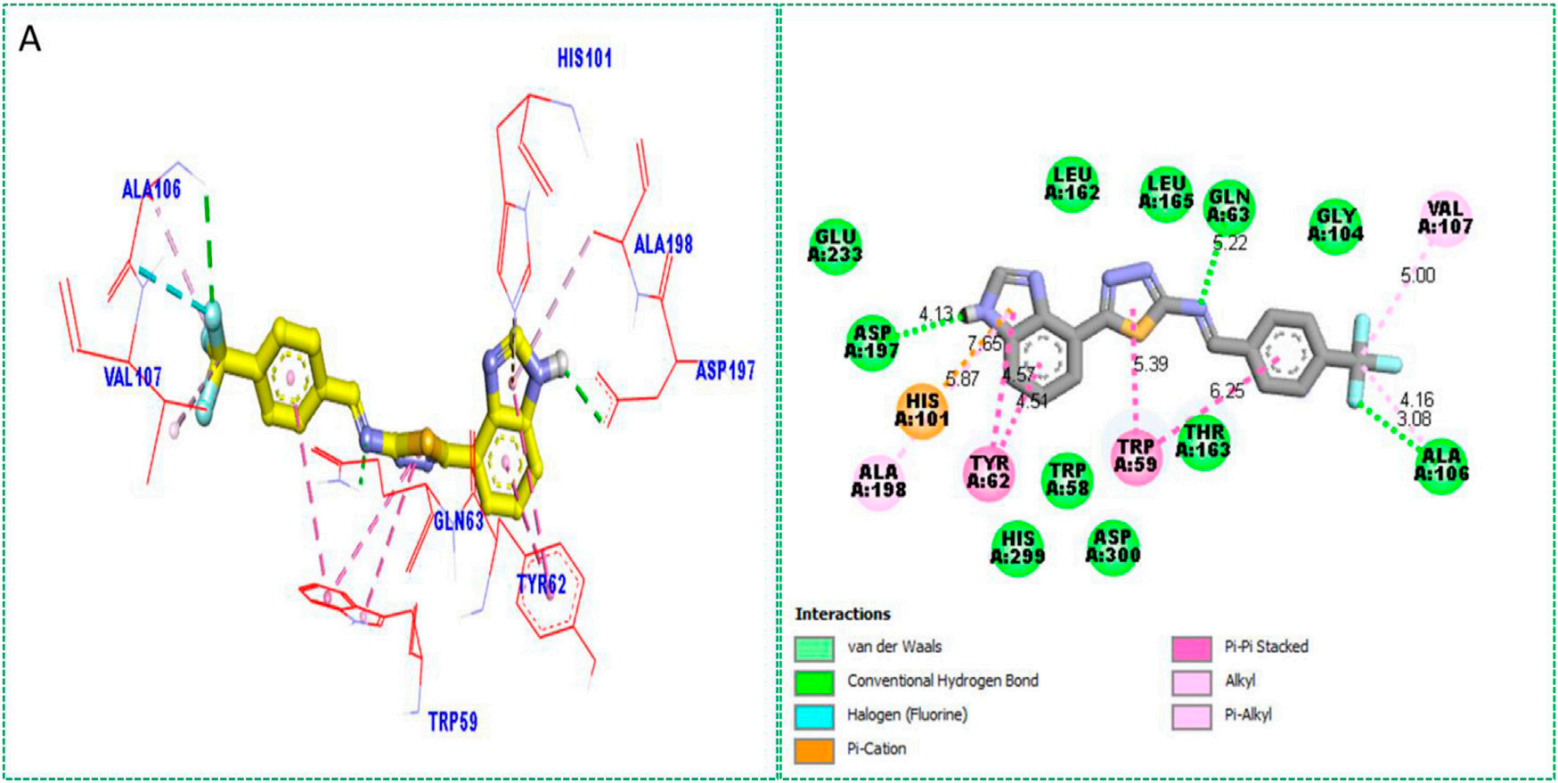
Protein–ligand interactions (PLI) of analog**-1** against α-amylase.

**FIGURE 4:**
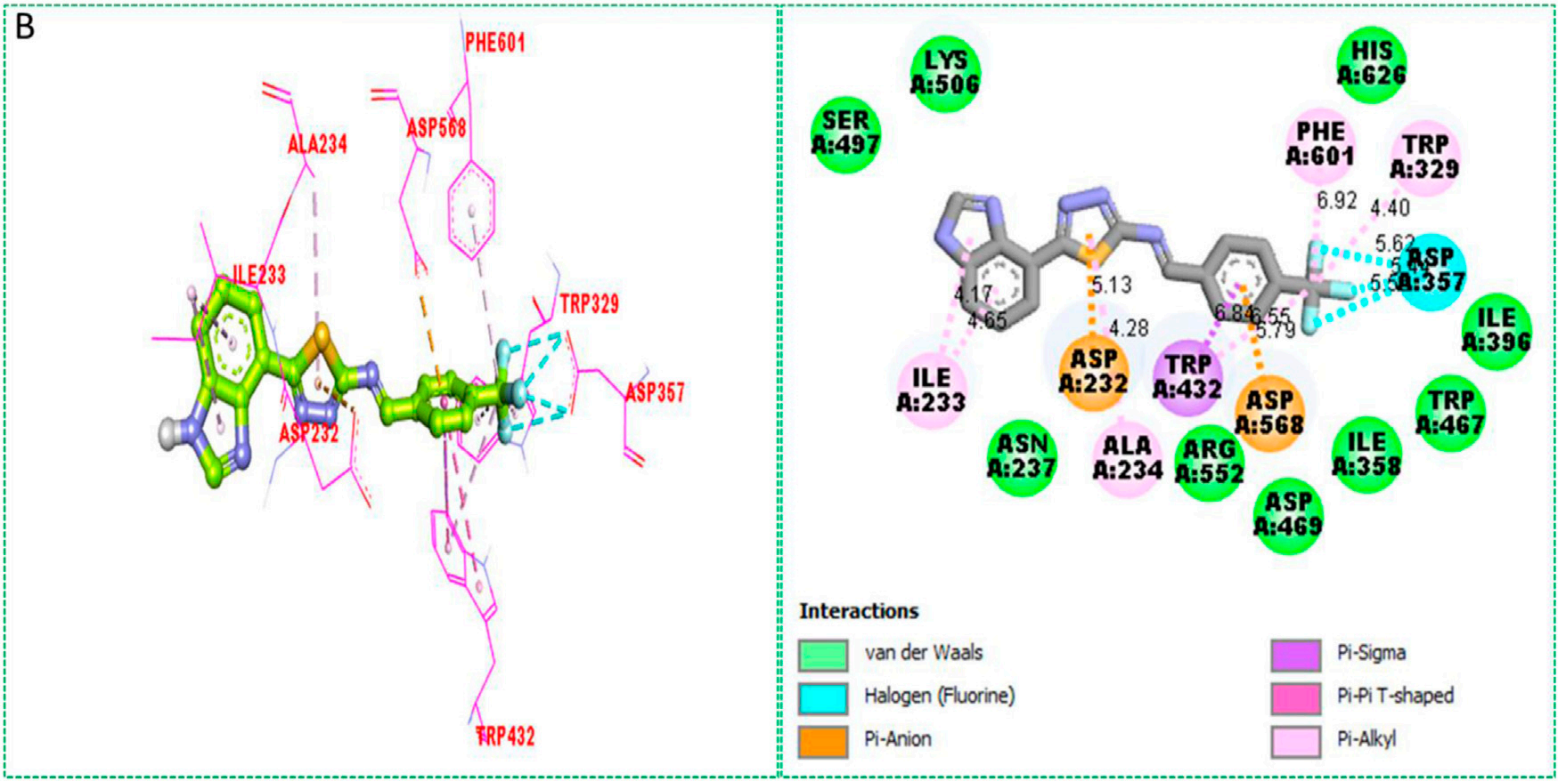
Protein–ligand interactions (PLI) of analog**-1** against α-glucosidase.

**FIGURE 5:**
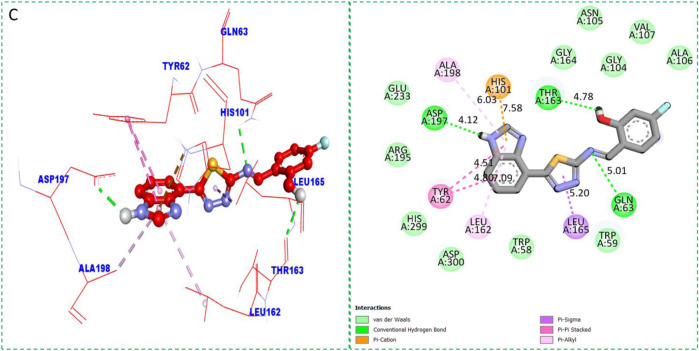
Protein–ligand interactions (PLI) of analog**-2** against α-amylase.

**FIGURE 6:**
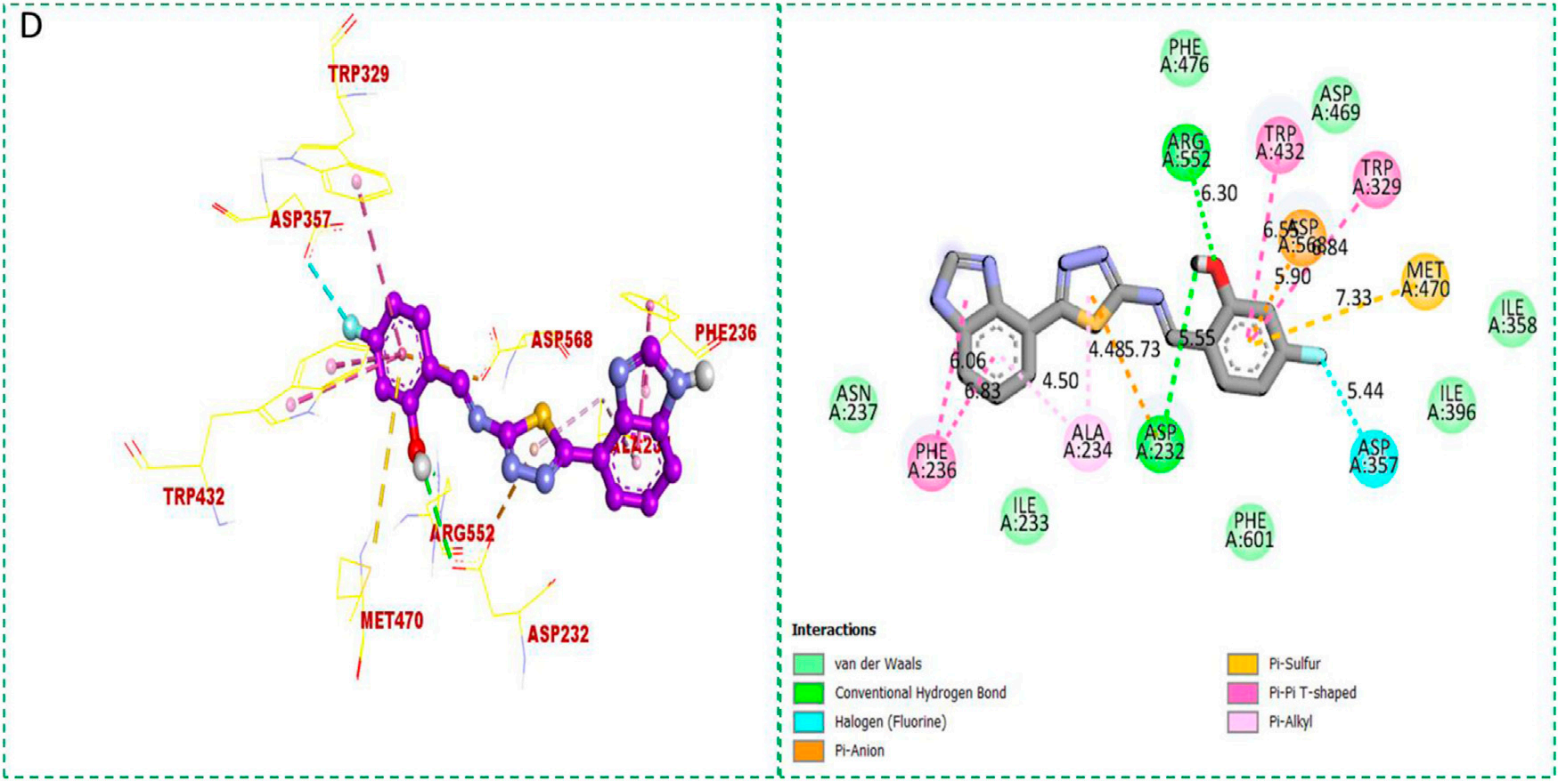
Protein–ligand interactions (PLI) of analog**-2** against α-glucosidase.

**FIGURE 7:**
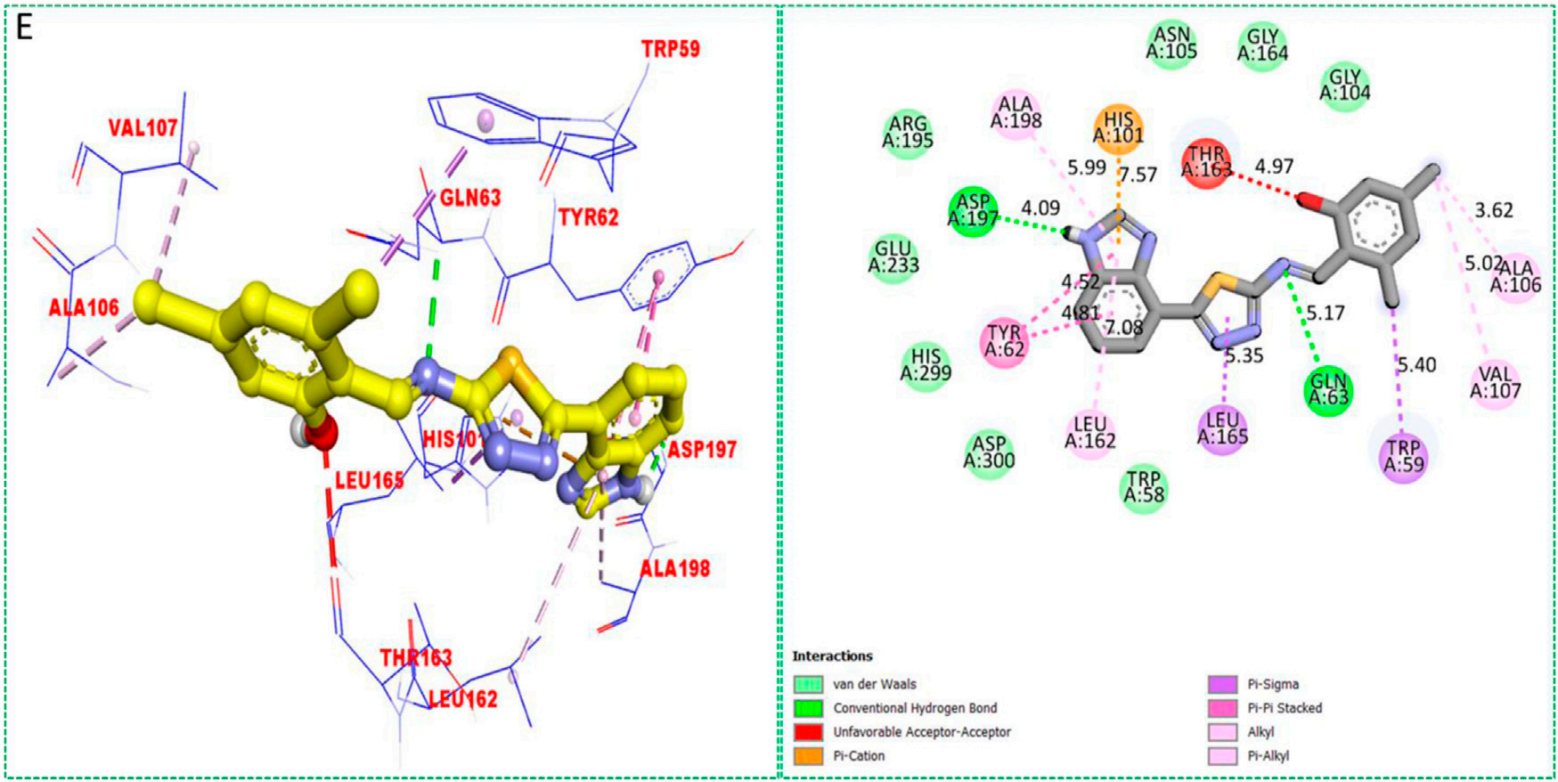
Protein–ligand interactions (PLI) of analog**-17** against α-amylase.

**FIGURE 8:**
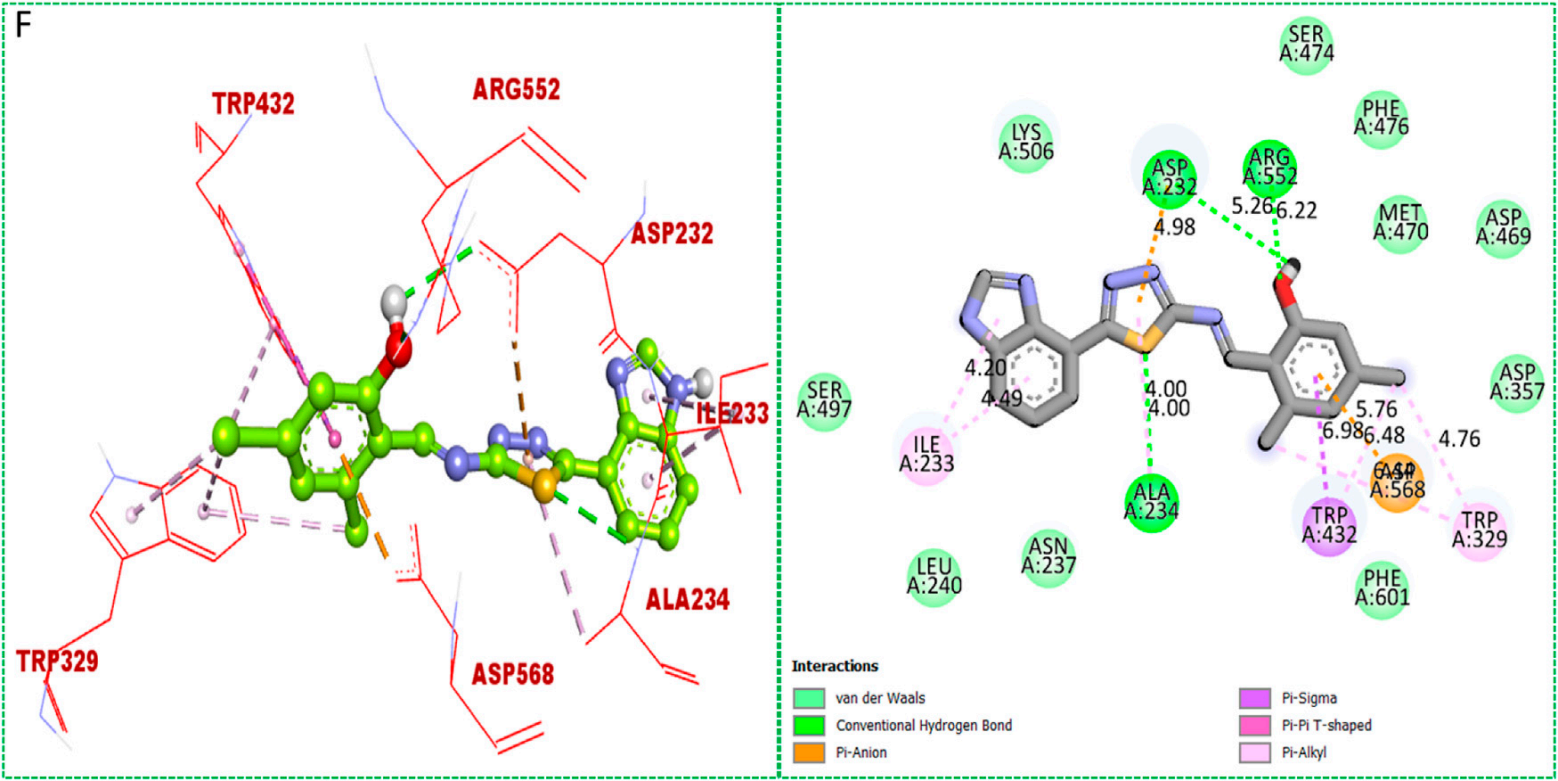
Protein–ligand interactions (PLI) of analog**-17** against α-glucosidase.

Based on the type of attached substituents, which either improve or diminish the interactive qualities, a molecular docking study can disclose the binding interaction of a molecule with the active sites of enzymes. A variety of tools, including Auto Dock Vina (1.5.7), Molecular Operational Environment (MOE-2015), and Discovery Studio Visualizer, were used to conduct molecular docking reQ1s3 earch (DSV-2021) (Kharb et al., 2012; Javid et al., 2018; Khan et al., 2022a; Khan et al., 2022b). Three processes were involved when carrying out the molecular docking studies. The first step protein and ligand, which were both downloaded from the RCSD protein data bank, and reducing energy in MOE. Next, the proteins and ligands were transferred to AutoDock in which polar hydrogen, Kollman, and Gasteiger charges were added and water molecules were removed from the protein. After completion, the PDBQT and the text formats of the protein, ligand, and their X, Y, and Z coordinates were saved, respectively. The final step involved specifying the location of the docking folder and performing a molecular docking investigation using the command prompt. Finally, the binding interaction was examined using DSV (Table 2) as 2D and 3D structures, as shown in Figure 3, Figure 4, Figure 5, Figure 6, Figure 7, and Figure 8.

**TABLE 2:** Binding interactions of selected molecules, showing the enzyme active residues, distances, and docking scores.

Compound	Receptor	Interaction	Distance	Docking score
Analog **1**(A) against α-amylase	ASP-A-197	H-B	4.13A°	−12.7
	HIS-A-101	Pi–cation	5.87A°	
	ALA-A-198	Pi–alkyl	7.65A°	
	TYR-A-62	Pi–pi stacked	4.51A°	
	TRP-A-59	Pi–Pi stacked	5.39A°	
	TRP-A-59	Pi–pi stacked	6.25A°	
	ALA-A-105	H-B	3.08A°	
	ALA-A-105	Alkyl	4.16A°	
	VAL-A-107	Pi–alkyl	5.00A°	
	GLN-A-63	H-B	5.22 A°	
Analog **1**(B) against α-glucosidase	ILE-A-233	Pi–alkyl	4.17A°	−11.6
	ASP-A-232	Pi–anion	5.13A°	
	ALA-A-234	Pi–alkyl	4.28A°	
	TRP-A-432	Pi–sigma	6.84A°	
	TRP-A-432	Pi–alkyl	6.55A°	
	ASP-A-568	Pi–anion	5.79A°	
	ASP-A-357	H-F	5.62A°	
	TRP-A-329	Pi–alkyl	4.40A°	
	PHE-A-601	Pi–aAlkyl	6.92A°	
Analog **2**(C) against α-amylase	TYR-A-62	Pi–pi stacked	4.51A°	−11.4
	LEU-A-162	Pi–alkyl	7.09A°	
	LEU-A-165	Pi–sigma	5.20A°	
	GLN-A-63	H-B	5.01A°	
	THR-A-163	H-B	4.78A°	
	HIS-A-101	Pi–cation	7.58A°	
	ALA-A-198	Pi–alkyl	6.03A°	
	ASP-A-197	H-B	4.12A°	
Analog **2**(D) against α-glucosidase	PHE-A-236	Pi–pi T-shaped	6.06A°	−10.2
	ALA-A-234	Pi–alkyl	4.50A°	
	ASP-A-232	H-B	5.55A°	
	ASP-A-232	Pi–anion	5.73A°	
	ASP-A-357	H-F	5.44A°	
	MET-A-470	Pi–sulfur	7.33A°	
	TRP-A-329	Pi–pi T-shaped	6.84A°	
	ASP-A-568	Pi–anion	5.90A°	
	TRP-A-432	Pi–anion	5.55A°	
	ARG-A-552	H-B	6.30A°	
Analog **17**(E) against α-amylase	TYR-A-62	Pi–pi stacked	4.95A°	−9.8
	LEU-A-162	Pi–alkyl	7.08A°	
	LEU-A-165	Pi-S	5.35A°	
	GLN-A-63	H-B	5.17A°	
	TRP-A-59	Pi-S	5.40A°	
	VAL-A-107	Pi-R	5.03A°	
	ALA-A-106	R	3.62A°	
	THR-A-163	Un-Acc.-Acc	4.97A°	
	HIS-A-101	Pi–cation	7.57A°	
	ALA-A-198	Pi-R	5.99A°	
	ASP-A-197	H-B	4.09A°	
Analog **17**(F) against α-glucosidase	ILE-A-233	Pi-R	4.20A°	−8.7
	ALA-A-234	H-B	4.00A°	
	ALA-A-234	Pi–anion	4.00A°	
	TRP-A-432	Pi–sigma	6.98A°	
	TRP-A-432	Pi–alkyl	6.48A°	
	ASP-A-568	Pi–anion	5.76A°	
	TRP-A-329	Pi–alkyl	6.44A°	
	ARG-A-552	H-B	6,22A°	
	ASP-A-232	H-B	5.26A°	
	ASP-A-232	Pi–anion	4.98A°	

The synthesized compounds with significant potential against α-amylase and α-glucosidase showed better interactions for the superimposed complex. Bonded functional groups at various positions on the aromatic ring provided analogs with a strong affinity. Trifluoro, nitro, and hydroxyl-containing molecules showed stronger hydrogen bonding. The attached substituents may have contributed to the good-to-poor interactions observed in most of the analogs, although **1, 2,** and **17** were the most active analogs, with the highest numbers of interactions. The grid dimension for this docking along the *X*, *Y*, and *Z*-axes were 12.037, −6.834, and 3.446, respectively. The size for all axes (X, Y, and Z) was 20.

## Conclusion

Benzimidazole-based thiadiazole analogs (**1–17)** were produced through a series of reactions using efficient and easy methods. The synthesized scaffolds were characterized using a variety of spectroscopic methods, including 1H-NMR, 13C-NMR, and HREI-MS, and they were tested against the enzymes α-glucosidase and α-amylase. The majority of them were discovered to have good to moderate inhibitory activity, however derivatives 1, 2, 5, 9, 11, and 17 were discovered to have superior activities against both α-amylase and α-glucosidase in comparison to the common medication acarbose. Among the evaluated analogs, analog2 (IC50 = 1.10 ± 0.10 and 2.10 ± 0.10 μM, respectively) was the most potent in the tested series. The α-amylase and α-glucosidase activity of a new class of benzimidazole-based thiadiazole derivatives was discovered. The synthetic derivativesdemonstrated strong correlates with the experimental results in molecular docking investigations. Active substanceswere identified as potential anti-diabetic leads based on their interactions with active site residues and their binding mechanisms. The novel, revolutionary structural hybrids of benzimidazole and thiadiazole moieties are new active leads and attractive possibilities for the development of anti-diabetic drugs due to the level of activity and docking studies they have shown.

## Data Availability

The original contributions presented in the study are included in the article/Supplementary Material. Further inquiries can be directed to the corresponding authors.
