# Supplementary material for: Synthesis, in vitro biological assessment, and molecular docking study of benzimidazole-based thiadiazole derivatives as dual inhibitors of α-amylase and α-glucosidase
Source: Front Chem. 2023 May 5;11:1125915. doi: 10.3389/fchem.2023.1125915 (PMC10196468; doi:10.3389/fchem.2023.1125915)
Supplement: Supplementary file 1 [file DataSheet1.docx]

**Supporting Information**

**Synthesis, In Vitro Biological Assessment and Molecular Docking Study of Benzimidazole-Based Thiadiazole Derivatives as dual Inhibitors of α-Amylase and α-Glucosidase**

Shoaib Khan^1^, Shahid Iqbal^2^*, Muhammad Taha^3^, Rafaqat Hussain^1^, Fazal Rahim^1^, Mazloom Shah^4^, Nasser S Awwad^5^, Hala A. Ibrahium^6,7^, Mohammed Issa Alahmdi^8^, Ayed A. Dera^9^, Hayat Ullah^10*^, Ali Bahadur^11^*, Samar O. Aljazzar^12^, Eslam B. Elkaeed^13^, Muhammad Rauf^14^

*^1^Department of Chemistry, Hazara University, Mansehra-21120, Pakistan.*

*^2^Department of Chemistry, School of Natural Sciences (SNS), National University of Science and Technology (NUST), H-12, Islamabad, 46000, Pakistan.*

*^3^Department of Clinical Pharmacy, Institute for Research and Medical Consultations (IRMC), Imam Abdulrahman Bin Faisal University, P.O. Box 31441, Dammam, Saudi Arabia.*

*^4^Department of Chemistry, Abbottabad University of Science and Technology (AUST) Abbottabad, Pakistan. ^5^Department of Chemistry, King Khalid University, P.O. Box 9004, Abha, 61413, Saudi Arabia.*

*^6^Department of Biology, Nuclear Materials Authority, P.O. Box 530, El Maadi, Egypt.*

*^7^Department of Semi Pilot Plant, Nuclear Materials Authority, P.O. Box 530, El Maadi, Egypt.*

*^8^Department of Chemistry, Faculty of Science, University of Tabuk, Tabuk- 71491, Saudi Arabia.*

*^9^Department of Clinical Laboratory Sciences, College of Applied Medical Sciences, King Khalid University, Abha, Saudi Arabia.*

*^10^Department of Chemistry, University of Okara, Okara-56300, Punjab, Pakistan.*

*^11^Department of Chemistry, College of Science and Technology, Wenzhou-Kean University, Wenzhou 325060, China.*

*^12^Department of Chemistry, College of Science, Princess Nourah bint Abdulrahman University, P.O. Box 84428, Riyadh 11671, Saudi Arabia.*

*^13^Department of Pharmaceutical Sciences, College of Pharmacy, AlMaarefa University, Riyadh 13713, Saudi Arabia.*

*^14^Department of Chemistry, School of Science, University of Management and Technology, Lahore 54770, Pakistan.*

****To whom corresponding should be addressed***

shahid.i14@yahoo.com (Shahid Iqbal) and abahadur@wku.edu.cn (A.B.)

***2.2 Spectral Analysis***

Following synthesized compounds (**1-17**) were characterized through HNMR, CNMR and HREI-MS and their detail interpretation are:

***2.2.1.*** ***(E)-N-(5-(1H-benzo[d]imidazol-4-yl)-1,3,4-thiadiazol-2-yl)-1-(4-(trifluoromethyl)phenyl)methanimine*** *(****1****)*

Yield 70%; ^1^HNMR (500 MHz, DMSO-*d_6_*): *δ*10.70 (s, 1H, NH), 8.43 (d, 2H, *J* = 7.6 Hz, Ar-H), 8.11 (s, 1H, -N=CH), 7.85 (t, 1H*, J* = 7.8 Hz, Benzimidazole-H), 7.82 (s, 1H*,* Benzimidazole-H), 7.71 (d, 2H*, J* = 7.6 Hz, Ar-H), 7.45 (d, 1H*, J* = 8.2 Hz, Benzimidazole-H), 7.26 (d, 1H, *J* = 7.0 Hz, Benzimidazole-H); ^13^CNMR (125 MHz, DMSO-d*_6_*) *δ*163.5, 158.8, 156.6, 154.3, 151.2, 145.0, 141.4, 136.5, 133.1, 132.9, 126.8, 124.3, 121.7, 120.4, 119.3, 112.5, 111.7;HREI-MS: m/z 373.5971; [M]^+^ Calcd for C_17_H_10_F_3_N_5_S; 373.4997.

***2.2.2. (E)-2-(((5-(1H-benzo[d]imidazol-4-yl)-1,3,4-thiadiazol-2-yl)imino)methyl)-5-fluorophenol*** *(****2****)*

Yield 60%; ^1^HNMR (500 MHz, DMSO-*d_6_*): *δ*11.52 (s, 1H, NH), 8.76 (d, 1H, *J* = 2.0Hz, Ar-H), 8.30 (s, 1H, -N=CH), 8.03 (t, 1H*, J* = 7.1 Hz, Benzimidazole-H), 7.95 (s, 1H*,* Benzimidazole-H), 7.82 (d, 1H*, J* = 7.9 Hz, Ar-H), 7.77 (d, 1H*, J* = 6.9 Hz, Ar-H), 7.63 (d, 1H*, J* = 7.4 Hz, Benzimidazole-H), 7.39 (d, 1H, *J* = 8.5 Hz, Benzimidazole-H); ^13^CNMR (125 MHz, DMSO-d*_6_*) *δ*178.5, 155.0, 154.6, 149.4, 147.6, 143.9, 139.3, 136.6, 133.8, 131.3, 129.7, 126.9, 125.1, 124.4, 122.1, 111.5; HREI-MS: m/z 339.9973; [M]^+^ Calcd for C_16_H_10_FN_5_OS; 339.9997.

***2.2.3. (E)-N-(5-(1H-benzo[d]imidazol-4-yl)-1,3,4-thiadiazol-2-yl)-1-(4-bromo-2-nitrophenyl)methanimine*** *(****3****)*

Yield 67%; ^1^HNMR (500 MHz, DMSO-*d_6_*): *δ*11.56 (s, 1H, NH),8.83 (d, 1H, *J* = 2.4Hz, Ar-H), 8.32 (s, 1H, -N=CH), 8.05 (t, 1H*, J* = 7.2 Hz, Benzimidazole-H), 7.99 (s, 1H*,* Benzimidazole-H), 7.84 (d, 1H*, J* = 6.9 Hz, Ar-H), 7.82 (d, 1H*, J* = 6.8 Hz, Ar-H), 7.66 (d, 1H*, J* = 6.4 Hz, Benzimidazole-H), 7.42 (d, 1H, *J* = 6.5 Hz, Benzimidazole-H); ^13^CNMR (125 MHz, DMSO-d*_6_*) *δ*188.5, 156.0, 155.6, 148.4, 146.6, 142.9, 140.3, 135.6, 132.8, 130.3, 128.7, 127.9, 126.1, 125.4, 125.1, 112.5; HREI-MS: m/z 427.2570; [M]^+^ Calcd for C_16_H_9_BrN_6_O_2_S; 427.2467.

***2.2.4. (E)-N-(5-(1H-benzo[d]imidazol-4-yl)-1,3,4-thiadiazol-2-yl)-1-(2 nitrophenyl)methanimine*** *(****4****)*

Yield 72%; ^1^HNMR (500 MHz, DMSO-*d_6_*): *δ*11.50 (s, 1H, NH), 8.36 (dd, 1H, *J* = 8.4, 1.9Hz, Ar-H), 8.11 (s, 1H, -N=CH), 8.05 (t, 1H*, J* = 7.5 Hz, Benzimidazole-H), 7.71 (s, 1H*,* Benzimidazole-H), 7.55 (dd, 1H*, J* = 7.0, 1.7 Hz, Ar-H), 7.49-7.46 (m, 1H*,* Ar-H), 7.40 (d, 1H*, J* = 7.4 Hz, Benzimidazole-H), 7.33-7.31 (m, 1H*,* Ar-H), 7.26 (d, 1H, *J* = 7.6 Hz, Benzimidazole-H); ^13^CNMR (125 MHz, DMSO-d*_6_*) *δ*159.8, 147.6, 144.3, 142.2, 141.0, 130.2, 129.5, 127.1, 125.9, 123.8, 121.3, 118.7, 116.4, 113.3, 112.0, 110.1; HREI-MS: m/z 350.0275; [M]^+^ Calcd for C_16_H_10_N_6_O_2_S; 350.0292.

***2.2.5. (E)-N-(5-(1H-benzo[d]imidazol-4-yl)-1,3,4-thiadiazol-2-yl)-1-(4-nitrophenyl)methanimine*** *(****5****)*

Yield 68%; ^1^HNMR (500 MHz, DMSO-*d_6_*): *δ*11.25 (s, 1H, NH), 8.64 (d, 2H, *J* = 6.6 Hz, Ar-H), 8.28 (s, 1H, -N=CH), 7.94 (t, 1H*, J* = 7.3 Hz, Benzimidazole-H), 7.89 (s, 1H*,* Benzimidazole-H), 7.83 (d, 2H*, J* = 7.9 Hz, Ar-H), 7.69 (d, 1H*, J* = 8.3 Hz, Benzimidazole-H), 7.32 (d, 1H, *J* = 7.0 Hz, Benzimidazole-H); ^13^CNMR (125 MHz, DMSO-d*_6_*) *δ*155.8, 154.6, 153.3, 150.2, 149.0, 147.4, 144.5, 141.1, 140.9, 136.8, 122.3, 120.7, 119.4, 117.3, 112.3, 111.2; HREI-MS: m/z 350.0175; [M]^+^ Calcd for C_16_H_10_N_6_O_2_S; 350.0092.

***2.2.6. (E)-N-(5-(1H-benzo[d]imidazol-4-yl)-1,3,4-thiadiazol-2-yl)-1-(p-tolyl)methanimine*** *(****6****)*

Yield 60%; ^1^HNMR (500 MHz, DMSO-*d_6_*): *δ*10.62 (s, 1H, NH), 8.34 (d, 2H, *J* = 7.1 Hz, Ar-H), 8.22 (s, 1H, -N=CH), 7.83 (t, 1H*, J* = 7.3 Hz, Benzimidazole-H), 7.76 (s, 1H*,* Benzimidazole-H), 7.55 (d, 2H*, J* = 8.4 Hz, Ar-H), 7.42 (d, 1H*, J* = 7.2 Hz, Benzimidazole-H), 7.18 (d, 1H, *J* = 7.8 Hz, Benzimidazole-H), 2.44 (s, 3H, CH3); ^13^CNMR (125 MHz, DMSO-d*_6_*) *δ*160.5, 157.8, 155.6, 153.3, 148.2, 146.0, 144.4, 143.5, 139.1, 136.9, 130.8, 127.3, 125.7, 123.4, 120.3, 114.5, 113.7, 40.5; HREI-MS: m/z 319.0527; [M]^+^ Calcd for C_17_H_13_N_5_S; 319.0543.

***2.2.7. (E)-N-(5-(1H-benzo[d]imidazol-4-yl)-1,3,4-thiadiazol-2-yl)-1-(o-tolyl)methanimine*** *(****7****)*

Yield 65%; ^1^HNMR (500 MHz, DMSO-*d_6_*): *δ*11.49 (s, 1H, NH), 8.51 (dd, 1H, *J* = 7.3, 2.1Hz, Ar-H), 8.15 (s, 1H, -N=CH), 8.01 (t, 1H*, J* = 7.2 Hz, Benzimidazole-H), 7.80 (s, 1H*,* Benzimidazole-H), 7.63 (dd, 1H*, J* = 6.6, 2.0 Hz, Ar-H), 7.56-7.54 (m, 1H*,* Ar-H), 7.50 (d, 1H*, J* = 7.8 Hz, Benzimidazole-H), 7.39-7.37 (m, 1H*,* Ar-H), 7.33 (d, 1H, *J* = 7.6 Hz, Benzimidazole-H), 2.94 (s, 3H, CH3); ^13^CNMR (125 MHz, DMSO-d*_6_*) *δ*153.8, 145.6, 143.3, 141.2, 138.0, 134.4, 131.5, 129.1, 125.9, 124.8, 123.3, 122.7, 121.4, 120.3, 115.5, 112.1, 45.4; HREI-MS: m/z 319.0727; [M]^+^ Calcd for C_17_H_13_N_5_S; 319.0643.

***2.2.8. (E)-N-(5-(1H-benzo[d]imidazol-4-yl)-1,3,4-thiadiazol-2-yl)-1-(m-tolyl)methanimine*** *(****8****)*

Yield 59%; ^1^HNMR (500 MHz, DMSO-*d_6_*): *δ*11.47 (s, 1H, NH), 8.47 (d, 1H, *J* = 2.1Hz, Ar-H), 8.15 (s, 1H, -N=CH), 8.01 (t, 1H*, J* = 7.2 Hz, Benzimidazole-H), 7.80 (s, 1H*,* Benzimidazole-H), 7.60 (d, 1H*, J* = 6.6 Hz, Ar-H), 7.54 (d, 1H*, J* = 8.0 Hz, Ar-H), 7.48 (d, 1H*, J* = 7.4 Hz, Benzimidazole-H), 7.36 (d, 1H, *J* = 7.8 Hz, Benzimidazole-H) 2.80 (s, 3H, CH3); ^13^CNMR (125 MHz, DMSO-d*_6_*) *δ* 153.6, 145.4, 143.6, 141.9, 138.3, 134.6, 131.8, 129.3, 125.7, 124.9, 123.1, 122.4, 121.1, 120.6, 115.7, 112.5, 45.7; HREI-MS: m/z 319.0627; [M]^+^ Calcd for C_17_H_13_N_5_S; 319.0543.

***2.2.9. (E)-N-(5-(1H-benzo[d]imidazol-4-yl)-1,3,4-thiadiazol-2-yl)-1-(4-fluorophenyl)methanimine*** *(****9****)*

Yield 63%; ^1^HNMR (500 MHz, DMSO-*d_6_*): *δ*10.70 (s, 1H, NH), 8.30 (d, 2H, *J* = 6.6 Hz, Ar-H), 8.10 (s, 1H, -N=CH), 7.81 (t, 1H*, J* = 7.5 Hz, Benzimidazole-H), 7.72 (s, 1H*,* Benzimidazole-H), 7.64 (d, 2H*, J* = 8.0 Hz, Ar-H), 7.40 (d, 1H*, J* = 8.1 Hz, Benzimidazole-H), 7.29 (d, 1H, *J* = 7.7 Hz, Benzimidazole-H); ^13^CNMR (125 MHz, DMSO-d*_6_*) *δ*167.5, 164.8, 161.6, 159.3, 156.2, 152.0, 151.4, 149.5, 142.1, 136.9, 133.8, 126.3, 122.7, 121.4, 120.3, 116.5, 113.7; HREI-MS: m/z 323.0275; [M]^+^ Calcd for C_16_H_10_FN_5_S; 323.0292.

***2.2.10. (E)-N-(5-(1H-benzo[d]imidazol-4-yl)-1,3,4-thiadiazol-2-yl)-1-(2,5-dimethoxyphenyl)methanimine*** *(****10****)*

Yield 68%; ^1^HNMR (500 MHz, DMSO-*d_6_*): *δ*10.74 (s, 1H, NH), 8.79 (d, 1H, *J* = 2.2 Hz, Ar-H), 8.34 (s, 1H, -N=CH), 8.31 (dd, 1H*, J* = 8.0, 2.2 Hz, Benzimidazole-H), 8.20 (dd, 1H, *J* = 8.6, 1.9 Hz, Benzimidazole-H), 8.04 (d, 1H, *J* = 6.4 Hz, Ar-H), 7.76 (d, 1H*, J* = 8.0 Hz, Ar-H), 7.68 (t, 1H*, J* = 6.6 Hz, Benzimidazole-H), 7.48 (s, 1H, Benzimidazole-H), 2.09 (s, 3H, CH3), 2.04 (s, 3H, CH3); ^13^CNMR (125 MHz, DMSO-d*_6_*) *δ*157.5, 149.6, 147.1, 144.4, 136.5, 131.9, 129.9, 129.8, 129.4, 129.2, 128.5, 123.4, 123.3, 122.8, 119.8, 115.8, 45.5, 42.0; HREI-MS: m/z 365.0274; [M]^+^ Calcd for C_18_H_15_N_5_O_2_S;365.0210.

***2.2.11. (E)-N-(5-(1H-benzo[d]imidazol-4-yl)-1,3,4-thiadiazol-2-yl)-1-(3-fluorophenyl)methanimine*** *(****11****)*

Yield 75%; ^1^HNMR (500 MHz, DMSO-*d_6_*): *δ*11.41 (s, 1H, NH), 8.40 (d, 1H, *J* = 1.8Hz, Ar-H), 8.07 (s, 1H, -N=CH), 8.00 (t, 1H*, J* = 7.8 Hz, Benzimidazole-H), 7.60 (s, 1H*,* Benzimidazole-H), 7.57 (d, 1H*, J* = 7.3 Hz, Ar-H), 7.49 (d, 1H*, J* = 8.0 Hz, Ar-H), 7.42 (d, 1H*, J* = 7.3 Hz, Benzimidazole-H), 7.30 (d, 1H, *J* = 7.8 Hz, Benzimidazole-H); ^13^CNMR (125 MHz, DMSO-d*_6_*) *δ*159.6, 143.4, 141.6, 140.9, 136.3, 133.6, 131.8, 126.3, 124.7, 123.9, 122.1, 121.4, 120.1, 119.6, 114.7, 111.5; HREI-MS: m/z 323.0675; [M]^+^ Calcd for C_16_H_10_FN_5_S; 323.0392.

***2.2.12. (E)-N-(5-(1H-benzo[d]imidazol-4-yl)-1,3,4-thiadiazol-2-yl)-1-(2-methoxyphenyl)methanimine*** *(****12****)*

Yield 69%; ^1^HNMR (500 MHz, DMSO-*d_6_*): 10.50 (s, 1H, NH), 8.40 (dd, 1H, *J* = 8.3, 2.1Hz, Ar-H), 8.10 (s, 1H, -N=CH), 8.03 (t, 1H*, J* = 7.7 Hz, Benzimidazole-H), 7.76 (s, 1H*,* Benzimidazole-H), 7.58 (dd, 1H*, J* = 7.6, 2.0 Hz, Ar-H), 7.53-7.51 (m, 1H*,* Ar-H), 7.45 (d, 1H*, J* = 7.0 Hz, Benzimidazole-H), 7.36-7.33 (m, 1H*,* Ar-H), 7.27 (d, 1H, *J* = 7.6 Hz, Benzimidazole-H), 3.30 (s, 3H, -OCH3); ^13^CNMR (125 MHz, DMSO-d*_6_*) *δ*151.8, 142.6, 141.3, 140.2, 135.0, 132.4, 130.5, 128.1, 122.9, 121.8, 120.3, 119.7, 117.4, 116.3, 114.5, 113.1, 44.4; HREI-MS: m/z 335.0472; [M]^+^ Calcd for C_17_H_13_N_5_OS;335.0492.

***2.2.13.* *(E)-N-(5-(1H-benzo[d]imidazol-4-yl)-1,3,4-thiadiazol-2-yl)-1-(3-methoxyphenyl)methanimine*** *(****13****)*

Yield 61%; ^1^HNMR (500 MHz, DMSO-*d_6_*): *δ*11.29 (s, 1H, NH), 8.28 (d, 1H, *J* = 2.4 Hz, Ar-H), 8.03 (s, 1H, -N=CH), 7.87 (t, 1H*, J* = 8.2 Hz, Benzimidazole-H), 7.74 (s, 1H*,* Benzimidazole-H), 7.48 (d, 1H*, J* = 7.6 Hz, Ar-H), 7.42 (d, 1H*, J* = 8.3 Hz, Ar-H), 7.38 (d, 1H*, J* = 7.8 Hz, Benzimidazole-H), 7.31 (d, 1H, *J* = 6.8 Hz, Benzimidazole-H), 3.41 (s, 3H, -OCH3); ^13^CNMR (125 MHz, DMSO-d*_6_*) *δ*153.2, 145.9, 143.4, 141.2, 138.0, 134.7, 131.4, 129.8, 125.9, 124.5, 123.4, 122.2, 121.3, 120.1, 115.6, 112.3, 45.1;HREI-MS: m/z 335.0372; [M]^+^ Calcd for C_17_H_13_N_5_OS;335.0292.

***2.2.14. (E)-N-(5-(1H-benzo[d]imidazol-4-yl)-1,3,4-thiadiazol-2-yl)-1-(4-chloro-2-nitrophenyl)methanimine*** *(****14****)*

Yield 64%; ^1^HNMR (500 MHz, DMSO-*d_6_*): *δ* 10.23 (s, 1H, NH), 8.50 (dd, 1H, *J* = 6.9, 2.0 Hz, Benzimidazole-H), 8.10 (dd, 1H, *J* = 6.9, 2.6Hz, Ar-H), 7.73 (d, 1H*, J* = 7.3 Hz, Ar-H), 7.47 (d, 1H*, J* = 6.9 Hz, Benzimidazole-H), 7.42 (t, 1H*, J* = 7.1 Hz, Benzimidazole-H), 7.39 (s, 1H, -N=CH), 7.30 (s, 1H, benzimidazole-H), 7.22 (d, 1H*, J* = 7.5 Hz, Ar-H); ^13^CNMR (125 MHz, DMSO-d*_6_*) *δ* 155.6, 147.5, 144.8, 144.2, 137.5, 135.5, 132.1, 129.4, 126.3, 125.4, 124.2, 123.2, 121.1, 116.8, 116.5, 112.3; HREI-MS: m/z 384.0239; [M]^+^ Calcd for C_16_H_9_ClN_6_O_2_S; 384.0260.

***2.2.15. (E)-N-(5-(1H-benzo[d]imidazol-4-yl)-1,3,4-thiadiazol-2-yl)-1-(2,3-dichlorophenyl)methanimine*** *(****15****)*

Yield 75%; ^1^HNMR (500 MHz, DMSO-*d_6_*): *δ*10.33 (s, 1H, NH), 8.48 (dd, 1H, *J* = 7.9, 1.7 Hz, Benzimidazole-H), 8.16 (dd, 1H, *J* = 6.7, 1.6 Hz, Ar-H), 7.66 (dd, 1H*, J* = 7.2, 2.0 Hz, Ar-H), 7.46 (d, 1H*, J* = 6.8 Hz, Benzimidazole-H), 7.40 (t, 1H*, J* = 7.3 Hz, Benzimidazole-H), 7.34 (s, 1H, -N=CH), 7.29 (s, 1H, Benzimidazole-H), 7.20 (t, 1H*, J* = 7.4 Hz, Ar-H); ^13^CNMR (125 MHz, DMSO-d*_6_*) *δ* 155.2, 147.9, 144.3, 144.8, 137.3, 135.7, 132.0, 129.6, 126.1, 125.9, 124.0, 123.3, 121.2, 116.5, 116.4, 112.0; HREI-MS: m/z 374.2476; [M]^+^ Calcd for C_16_H_9_Cl_2_N_5_S; 373.2291.

***2.2.16. (E)-N-(5-(1H-benzo[d]imidazol-4-yl)-1,3,4-thiadiazol-2-yl)-1-(4-bromophenyl)methanimine*** *(****16****)*

Yield 67%; ^1^HNMR (500 MHz, DMSO-*d_6_*): *δ*10.82 (s, 1H, NH), 8.26 (d, 2H, *J* = 7.0 Hz, Ar-H), 8.17 (s, 1H, -N=CH), 7.83 (t, 1H*, J* = 7.4 Hz, Benzimidazole-H), 7.76 (s, 1H*,* Benzimidazole-H), 7.67 (d, 2H*, J* = 7.0 Hz, Ar-H), 7.50 (d, 1H*, J* = 8.1 Hz, Benzimidazole-H), 7.35 (d, 1H, *J* = 7.7 Hz, Benzimidazole-H); ^13^CNMR (125 MHz, DMSO-d*_6_*) *δ*166.5, 162.8, 160.6, 157.3, 155.2, 151.0, 150.4, 148.5, 145.1, 135.9, 132.8, 124.3, 121.7, 120.4, 119.5, 115.5, 114.7; HREI-MS: m/z 382.0215; [M]^+^ Calcd for C_16_H_10_BrN_5_S; 382.0150.

***2.2.17. (E)-N-(5-(1H-benzo[d]imidazol-4-yl)-1,3,4-thiadiazol-2-yl)-1-(4-bromophenyl)methanimine*** *(****17****)*

Yield 73%; ^1^HNMR (500 MHz, DMSO-*d_6_*): *δ* 10.75 (s, 1H, NH), 9.44 (s, 1H, OH), 8.26 (s, 1H, Ar-H), 8.16 (s, 1H, -N=CH), 7.81 (t, 1H*, J* = 8.0 Hz, Benzimidazole-H), 7.73 (s, 1H*,* Benzimidazole-H), 7.50 (s, 1H*,* Ar-H), 7.45 (d, 1H*, J* = 7.1 Hz, Benzimidazole-H), 7.30 (d, 1H, *J* = 7.4 Hz, Benzimidazole-H); ^13^CNMR (125 MHz, DMSO-d*_6_*) *δ*164.5, 161.8, 161.6, 157.3, 155.8, 151.9, 150.6, 148.4, 145.2, 135.5, 132.0, 124.2, 121.4, 120.7, 119.4, 115.2, 114.1; HREI-MS: m/z 382.0298; [M]^+^ Calcd for C_16_H_9_Cl_2_N_5_OS; 382.0237.


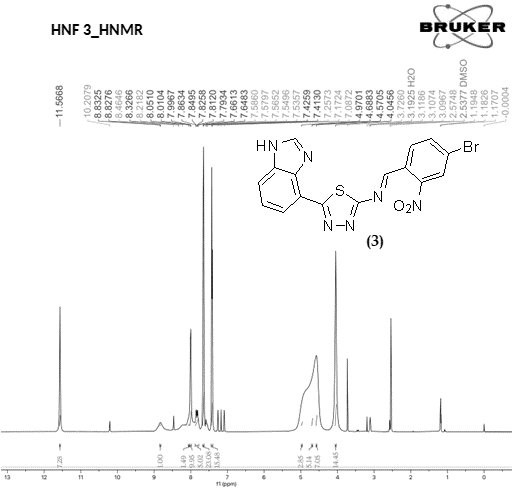


**Figure-9:**Represent HNMR of analog 3


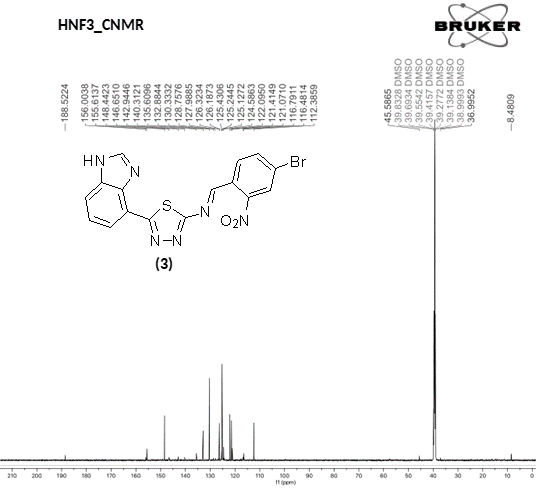


**Figure-10:**Represent CNMR of analog 3


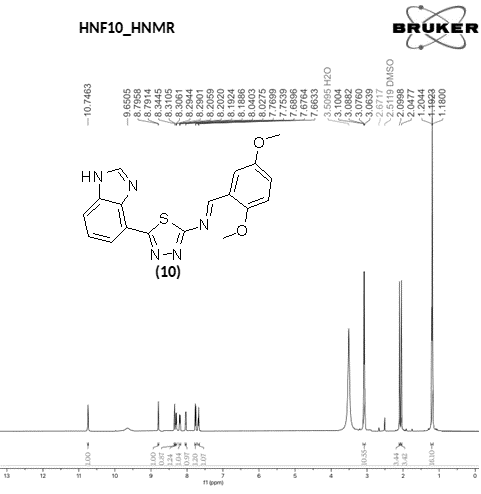


**Figure-11:**Represent HNMR of analog 10


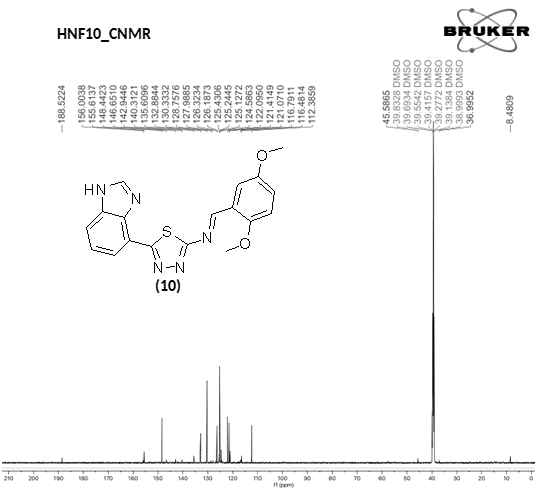


**Figure-12:**Represent CNMR of analog 10


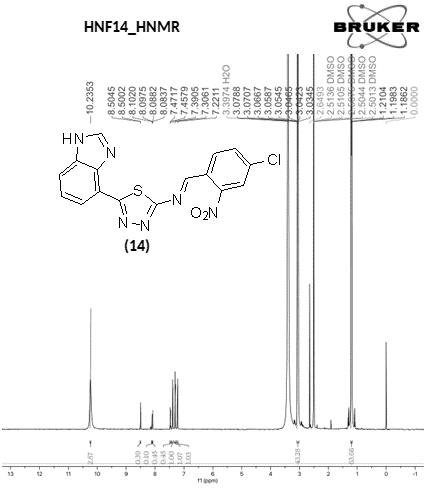


**Figure-13:**Represent HNMR of analog 14


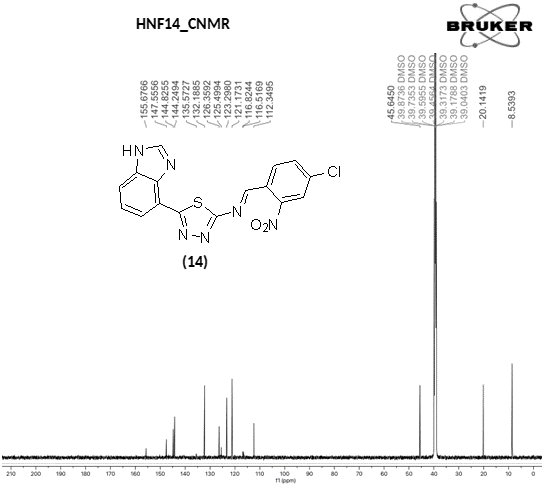


**Figure-14:**Represent CNMR of analog 14

**3.2.1 Molecular docking protocol**

**3.2.1.1*α-amylase inhibitory assay protocol***

20µL of α -amylase solution (0.5 mg/ml) was added to phosphate buffer (pH 6.9, 0.02 M, 200 µL). 250 μL of test samples (10-50 µg/mL) was added, to all the above solutions and kept for incubation for 10 min.1% starch solution (200µL) was further added and again kept for incubation for 10 min at a temperature of 25°C. Termination of the reaction was affected by the addition of 400 μL of 3, 5-dinitrosalicylic acid (DNS) reagent. Lastly, it was incubated in water (at 70°C, 5 min). The absorbance was recorded using an ELISA microplate reader at 540 nm. Acarbose was served as the standard. All the reactions were carried out in triplicates. The reaction mixture, devoid of the test sample, served as control. The following formula was employed to calculate percentage inhibition. [1].

% Inhibition = [(Ac – As) /Ac] × 100 Ac-Control absorbance, As-Standard absorbance

**3.2.1.2*α-glucosidase inhibitory assay protocol***

10 µl of the α-glucosidase enzyme, 20 µl of test samples (10-50 µg/ml) and 50 µl, of 0.1M phosphate buffer (pH=6.8), were incubated at 37°C for 15 min in a 96-well plate. 20 µl of p-nitrophenyl—D-glucopyranoside solution was further added as a substrate and again incubated for 20 min at 37 oC. Sodium carbonate (50 µl of 0.1M) was added to terminate the reaction. P-nitrophenol was liberated, during the reaction which was measured at 405nm using an ELISA microplate reader. Acarbose served as the standard. A control was prepared under similar conditions by omitting test samples. All the experiments were carried out in triplicates. The percentage inhibition was calculated by the formula. The IC50 values are tabulated in table-7 [1].

% Inhibition = [(Ac – As) /Ac] × 100 Ac-absorbance for control, As-absorbance for standard.

**3.2.1.3*Molecular docking assay protocol***

In this study the synthesized compounds were analyzed against α-amylase and α-glucosidase enzyme. In the first step protein was prepared by using DSV by removing water molecules and already present ligand were removed save both the target protein as well as prepared ligand in PDB format. The process was further carried out in auto dock in which polar hydrogen and Kollman and Gasteiger charges were added to protein. Selected ligand was also prepared done by using torsion tree to detect root. Moreover, configuration file was generated along with X, Y and Z axis save both ligand and protein in PDBQT format in the same docking folder. At the end command prompt was used to generate varied poses of ligand thus, 9 different poses were obtained in PDBQT format. The dock protein and ligand were then open in DSV to identify the binding interaction of ligand with active sites of enzyme [2].

**References**

[1] Srinivasa, M. G., Aggarwal, N. N., Gatpoh, B. F. D., Shankar, M. K., Byadarahalli Ravindranath, K., Gurubasavaraj Veeranna, P., ... & Bistuvalli Chandrashekarappa, R. (2022). Identification of benzothiazole‐rhodanine derivatives as α‐amylase and α‐glucosidase inhibitors: Design, synthesis, in silico, and in vitro analysis. *Journal of Molecular Recognition*, 2959.

[2] Khan, S., Ullah, H., Rahim, F., Nawaz, M., Hussain, R., & Rasheed, L. (2022). Synthesis, in vitro α-amylase, α-glucosidase activities and molecular docking study of new benzimidazole bearing thiazolidinone derivatives. *Journal of Molecular Structure*, 133812.
